# Early Responses of Natural Killer Cells in Pigs Experimentally Infected with 2009 Pandemic H1N1 Influenza A Virus

**DOI:** 10.1371/journal.pone.0100619

**Published:** 2014-06-23

**Authors:** Hilde Forberg, Anna G. Hauge, Mette Valheim, Fanny Garcon, Alejandro Nunez, Wilhelm Gerner, Kerstin H. Mair, Simon P. Graham, Sharon M. Brookes, Anne K. Storset

**Affiliations:** 1 Department of Laboratory Services, Norwegian Veterinary Institute, Oslo, Norway; 2 Virology Department, Animal Health and Veterinary Laboratories Agency, Addlestone, United Kingdom; 3 Pathology Department, Animal Health and Veterinary Laboratories Agency, Addlestone, United Kingdom; 4 Department of Pathobiology, University of Veterinary Medicine Vienna, Vienna, Austria; 5 Department of Food Safety and Infection Biology, Norwegian University of Life Sciences, Oslo, Norway; Beth Israel Deaconess Medical Center, Harvard Medical School, United States of America

## Abstract

Natural killer (NK) cells are important players in the innate immune response against influenza A virus and the activating receptor NKp46, which binds hemagglutinin on the surface of infected cells, has been assigned a role in this context. As pigs are natural hosts for influenza A viruses and pigs possess both NKp46^−^ and NKp46^+^ NK cells, they represent a good animal model for studying the role of the NKp46 receptor during influenza. We explored the role of NK cells in piglets experimentally infected with 2009 pandemic H1N1 influenza virus by flow cytometric analyses of cells isolated from blood and lung tissue and by immunostaining of lung tissue sections. The number of NKp46^+^ NK cells was reduced while NKp46^−^ NK cells remained unaltered in the blood 1–3 days after infection. In the lungs, the intensity of NKp46 expression on NK cells was increased during the first 3 days, and areas where influenza virus nucleoprotein was detected were associated with increased numbers of NKp46^+^ NK cells when compared to uninfected areas. NKp46^+^ NK cells in the lung were neither found to be infected with influenza virus nor to be undergoing apoptosis. The binding of porcine NKp46 to influenza virus infected cells was verified in an *in vitro* assay. These data support the involvement of porcine NKp46^+^ NK cells in the local immune response against influenza virus.

## Introduction

Natural killer (NK) cells are innate lymphocytes that provide early protection against a number of viral infections and are thought to participate in the early defence against influenza virus [1], [2]. Human NK cells recognize influenza virus infected cells through the activating NK cell receptor NKp46/NCR1 *in vitro* [3], [4], but their importance *in vivo* is still poorly documented. Clinical cases of human influenza have shown that the population of NK cells in the blood is reduced. It has been proposed that NK cells migrate from the blood into the lungs [5]–[7] where they may become infected and killed by the influenza virus [8], [9]. Supporting this theory are findings of increased numbers of NK cells in the lungs of influenza infected mice [10]–[12]. Little is known about the function of NK cells during an influenza virus infection. However, NK cells kill influenza virus infected cells from mice and humans *in vitro* [3], [4], [13]–[15]. They are also important early producers of antiviral cytokines such as interferon gamma (IFN-γ) and tumour necrosis factor (TNF), which have been associated with the acute stages of influenza virus infection in both humans and swine. [16]–[20].

NK cells in swine blood are identified as CD3^−^CD8α^+^ cells [21], and a recent study has found that only about half of the NK cell population in the blood of swine express NKp46 [22]. NKp46^−^ NK cells show a similar ability to kill target cells but produce less IFN-γ as compared to NKp46^+^ NK cells. In liver and spleen, a third NK cell population with a higher NKp46 expression and a dim or negative CD8α expression has been described [22]. These CD8^−/dim^NKp46^high^ NK cells appear to represent a more activated state [23].

To better understand infectious disease and in particular zoonotic diseases such as influenza, it is important to undertake studies in natural hosts [24]. In addition to being a natural host to influenza, swine are anatomically and physiologically similar to humans and respond to an influenza virus infection in much the same manner as humans [17]–[20], [25]–[27]. Swine are therefore proposed to serve as a valuable model for studying the pathogenesis of respiratory diseases, including influenza, as well as the immune response against it [1], [28]–[30]. They also present a unique model for studying the role of NK cells expressing the NKp46 receptor *in vivo*, since they have both NKp46^−^ and NKp46^+^ NK cells [22].

In spring 2009 a novel influenza A virus, A (H1N1) pdm09, of putative swine origin with gene segments derived from human, swine and avian influenza viruses, spread in the human population causing a pandemic. Although this virus acquired the ability to transmit efficiently among humans, it also retained its ability to infect and be maintained in pigs [25], [31], [32]. The 2009 pandemic influenza A virus is now circulating in both humans and swine in most countries worldwide [32]. Most often, this virus causes a benign and self-limiting respiratory disease in humans and pigs, but severe cases have been reported in humans [26]. Since it occurs naturally in both species, the 2009 pandemic influenza A virus is well suited for comparative studies of influenza in humans and swine.

There are few reports on the role of NK cells in swine during influenza virus infection, and the results are conflicting [29], [33]. Furthermore, no studies have distinguished between NKp46^−^ and NKp46^+^ NK cells. Here, we provide evidence that NKp46^+^ NK cells are recruited from the blood to infected parts of the lungs in swine inoculated with the 2009 pandemic influenza virus. Moreover, the NKp46^+^ cells in the lungs are not infected by influenza virus and do not undergo apoptosis. This is the first report on the role of NKp46^+^ NK cells in pigs infected with influenza A virus and further demonstrates the value of the pig as a model for clinical disease in humans.

## Material and Methods

### Ethical statement

The project was approved by the Animal Health and Veterinary Laboratories Agency Ethics Committee, and all procedures were conducted in accordance with the UK Animals (Scientific Procedures) Act 1986 under Project License Permit Number 70/7062.

### Animals and virus

In two independent challenge experiments, six week-old-pigs were sourced from a high health status UK herd of the large white cross breed and tested negative for influenza A virus by Matrix real-time RT-PCR (rRT-PCR) [34] and pre-existing antibodies (subtypes H1N1, H1N2, H3N2 and H1N1pdm09) by hemagglutinin inhibition test [35]. The animals were kept in approved facilities and were closely monitored throughout the experiments. Two virus isolates were used in the infections; A/Hamburg/05/2009 (Ham) and A/Hamburg/05/2009-e (Ham-e) [36].

### Experimental infection and sampling

The pigs were inoculated via intranasal aerosols with 10^6^ TCID_50_ in 2 ml per nostril using a mucosal atomization device (MAD 300, Wolfe Tory Medical). In the first study, 12 pigs were inoculated with a 1∶1 mixture of the influenza A (H1N1) pdm09 225D (Ham) and 225G (Ham-e) variants. 12 pigs were mock-inoculated with similarly diluted clean cell culture medium and included as control animals. Two pigs from each group were sedated and euthanized by intravenous injection of barbiturates prior to post-mortem examination and sampling at days 1–5 and 8 post-infection (pi). In the second study, 12 pigs were inoculated solely with the 225G (Ham-e) variant. An untreated group consisting of 13 pigs was included as controls. Ten control animals were euthanized whilst three remaining pigs were used for blood analysis only. Four control pigs were euthanized prior to the experiment, while four infected and two control pigs were euthanized daily from day 1 to 3 pi.

**Figure 1:**
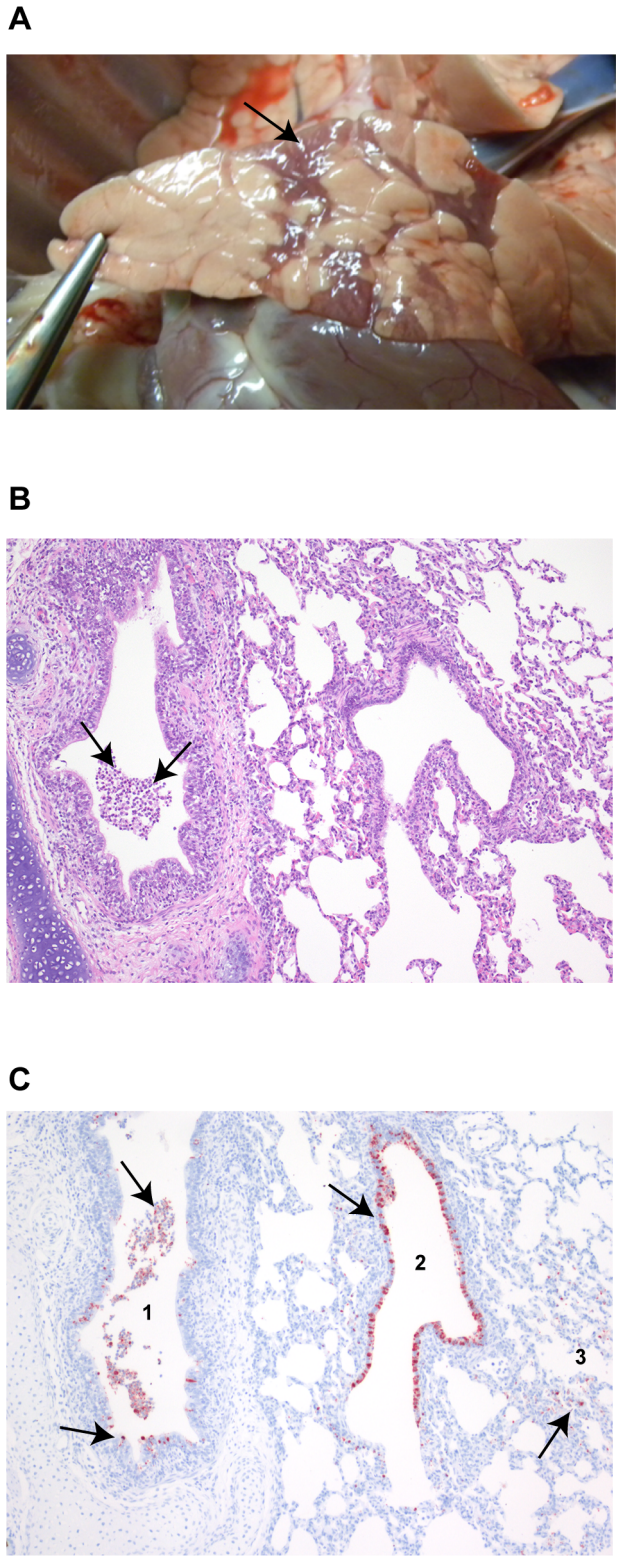
Bronchointerstitial pneumonia in pigs infected with influenza A virus. Pathological changes were recorded in the lungs of twelve pigs inoculated with influenza A virus on days 1–5 and 8 pi (*n* = 2 per day). (**A**) Macroscopic lesions were most prominent on day 3 pi; representative lesions are shown (arrow). (**B**) Histological changes on day 1 pi; representative lesions are shown. Leucocytes and cell debris (arrows) in the lumen of a bronchus. Hematoxylin and eosin, 100x. (**C**) Influenza A virus NP positive staining (arrows) in luminal cells and in the epithelial lining of a bronchus (1), a bronchiole (2) and in the pneumocytes of the parenchyma (3) in the same animal as shown in (C), serial sections (NP stained with AEC and counterstained with hematoxylin, 100x).

For both studies, animals were randomly allocated to each of the two groups and the day of post-mortem examination. Heparinized blood was collected from all animals on each sampling day. At post-mortem, systematic recording of macroscopic findings and blinded estimation of the gross lung lesion score was carried out as previously described [37], as well as the collection of tissues for further analysis. From the lungs, samples were taken from the cranial, middle, caudal and accessory lung lobes from the right side. Macroscopically affected areas (Fig. 1A) were chosen if present.

### Clinical observations and virological monitoring

Pigs were recorded daily for signs of clinical disease. For detection of viral RNA, total RNA was extracted from lung tissue homogenate supernatant and matrix gene rRT-PCR was performed as previously described [34]. Presence of virus was verified by virus re-isolation and titration in MDCK cells [35].

### Histopathology and immunohistochemistry

Tissues for histopathological examination and immunohistochemical analysis were collected from the nasal concha, trachea, lungs, lymph node draining the nasal cavity (LN retropharyngeales), lung lymph node (LN tracheabronchiale), liver, kidney, spleen and encephalon. Tissue was prepared and stained with hematoxylin and eosin for histopathological evaluation or used for immunohistochemical detection of influenza A virus nucleoprotein (NP) by the avidin-biotin-peroxidase complex method as previously described [25]. Influenza A virus NP was only detected in the respiratory tract and associated lymph nodes.

### Isolation of porcine mononuclear cells

For the first study, mononuclear cells from the blood, spleen, liver and lymph nodes draining the spleen (LN lienales), liver (LN hepaticiseuportales), lung (LN tracheobronchiale) and nasal cavities (LN retropharyngeales) were analysed by flow cytometry. For the second study, cells from the four lung lobes were additionally included, while cells from the spleen and LN lineales were omitted.

Pheriperial blood mononuclear cells (PBMC's) were isolated from heparinized blood by density gradient centrifugation using Ficoll-Paque Premium with a density of 1,077 g/ml (GE Healthcare). Tissue samples from all organs were homogenized using disposable scalpels. Mononuclear cells from liver and spleen were isolated by forcing homogenized tissue samples through a cell strainer (70 µm, BD Biosciences) before purification by density gradient centrifugation. Homogenized tissue from the lymph nodes was forced through a cell strainer and further sifted through a cotton wool filter to remove any remaining tissue debris. Mononuclear cells from lung tissue were isolated by enzymatic degradation before density gradient centrifugation. Briefly described, homogenized tissue was incubated for 60 min at 37°C in Gibco RPMI1640 Medium with Hepes (Invitrogen) with 2% FBS, gentamicin, 300 U/ml collagenase type 1 (Worthington) and 500 U/ml DNase (Sigma Aldrich). The processed tissue was then sifted through a cotton wool filter before mononuclear cells were isolated by density gradient centrifugation. Isolated mononuclear cells from the lungs not used in the flow cytometric analysis from infected animals were frozen in Gibco Recovery Cell Culture Freezing Medium (Invitrogen) at a concentration of 5×10^6^ cells per ml media and stored at −80°C for later use.

### Incubation of isolated mononuclear cells for IFN-γ staining

In study 2, cells isolated from lung and lung lymph nodes were analysed for intracellular IFN-γ expression by flow cytometry. Cells isolated from liver were included as controls. Cells (5×10^6^per well in a 24 well plate) from each tissue were incubated in 500 µl Gibco RPMI 1640 Medium Hepes (Invitrogen) with 10% FBS, gentamicin and 1 µl/ml BD Golgi Plug (BD Biosciences) for 4 hours at 37°C. All samples were set up in parallel; one of the two wells was incubated with 100 IU/ml recombinant porcine IL-2 (BioSupply) while the other well was incubated without cytokine. No difference was found between cells incubated with or without IL-2, and cells incubated with IL-2 were chosen for subsequent analysis.

### Flow cytometry and antibodies

In the second study, complete blood counts were obtained by flow cytometry. Whole blood was incubated for 10 min with CD45-FITC mAb (IgG1, clone K252-1E4, AbD Serotec), before 10 min incubation with FACS Lysing solution (BD Biosciences) at room temperature to lyse erythrocytes and fix leukocytes. Samples were diluted in PBS and cell counts were obtained on a volumetric flow cytometer (MACSQuant, Miltenyl Biotec). Lymphocytes were gated as CD45^high^ cells with a low side scatter and granulocytes as CD45^dim^ cells with a high side scatter.

For further analysis, isolated mononuclear cells were used in a concentration of 2.5×10^6 ^cells/ml in 200 µl. Cells were incubated with LIVE/DEAD Fixable Near-IR Dead Cell Kit (Invitrogen) for 10 min at room temperature, and washed once in Dulbecco's PBS without Mg^2+^ and Ca^2+^ (Invitrogen) supplemented with 10% porcine plasma and.09% sodium azide (FCM buffer) prior to staining. The FCM buffer was also used for all washing steps during the surface staining procedure.

Cells from blood and all tissues were stained with antibodies against CD3, CD8α and NKp46 in combination with CD25 or Ki-67. Stimulated cells from lung, lung lymph nodes and liver were additionally stained with antibodies against CD3, CD8α and NKp46 in combination with IFN-γ. For surface staining, primary antibodies and secondary reagents were used at previously determined optimal concentrations and incubated for 20 min on ice in the dark: CD3-eFluor450 (IgG1, clone PPT3), CD8α (IgG2a, clone 11/295/33), CD8α-AlexaFluor 647 (IgG2a, clone 11/295/33), NKp46-biotin (IgG1, clone VIV-KM1), CD25-AlexaFluor 647 (IgG1, clone 3B2), goat anti-mouse IgG2a-AlexaFluor 488 (Invitrogen), Streptavidin-AlexaFluor 647 (Invitrogen) and Streptavidin-PE (eBioscience). Wells were washed three times following incubation with primary antibodies and twice following incubation with secondary reagents. For intracellular staining, cells were fixed and permeabilized using the BD Cytofix/Cytoperm Fixation/Permeabilization Solution Kit (BD Biosciences), followed by intracellular staining according to the manufacturer's instruction using CD3-Pacific Blue mAb (IgG1, clone CD3-12, AbDSerotec) and Ki-67-FITC mAb (IgG1, clone B56, BD Biosciences) or IFN-γ-PE mAb (IgG1, clone P2G10, BD Biosciences). Appropriate secondary and isotype controls were included. Where not specified, antibodies were produced in-house from hybridoma cultures, purified and biotinylated or conjugated to fluorochromes as described elsewhere [38]. CD3-eFluor450 was obtained by a custom conjugation (eBioscience). Acquisition was performed on a MACSQuant Analyser flow cytometer (Miltenyi Biotec) and data was analysed using MACSQuantify software. A total of 100 000 cells were analysed when possible. Dead cells were excluded and lymphocytes were gated individually according to forward/side scatter characteristics. CD3^−^ cells were gated according to the expression of CD8α and NKp46, and defined as NKp46^−^ or NKp46^+^ NK cells in blood and as NKp46^−^, NKp46^int^ or NKp46^high^ NK cells in tissues.

### Immunofluorescence staining of lung tissue sections

Pulmonary samples were blocked to maximum thickness of 0.5 cm, embedded in cryomoulds containing Tissue-TEK O.C.T compound (Sakura Finetek Europe) and snap frozen by immersion in cooled isopentane over dry ice followed by storage at −80°C. The samples were cut on a cryostat, and 7 µm sections were placed on Polysine glass slides (Menzel GmbH & Co Kg, Braunschweig) and stored at −70°C until staining. A triple indirect fluorescence technique [39] with minor modifications was performed to simultaneously localize influenza A virus infected cells and NKp46^+^ cells in combination with either cytokeratin^+^ or caspase-3^+^ cells. Briefly, frozen sections were air-dried, fixed in acetone for 10 min before incubation with 20% BSA for 10 min to reduce non-specific binding. Antibodies against influenza A virus NP (IgG2a, clone F8, Abnova), NKp46 (IgG1, clone VIV-KM1, produced in-house), cytokeratin 8 (rabbit polyclonal, Antibodies-online) or caspase-3 (rabbit polyclonal, ACTIVE Caspase-3, Promega) were diluted in 2.5% BSA and simultaneously added to the sections. Incubations were carried out for 1 hour at room temperature. Secondary reagents used were goat anti-mouse IgG2a-AlexaFluor 546, goat anti-mouse IgG1-AlexaFluor 488 and goat anti-rabbit AlexaFluor 405. The sections were coverslipped with Fluka Polyvinyl alcohol mounting medium with DABCO antifading (Sigma-Aldrich). The controls included the omission of primary antibodies and known influenza A virus negative sections.

### Distribution and quantification of NKp46^+^ cells in lung tissue sections

The immunofluorescence stained lung tissue sections were examined with a Nikon Eclipse 80i microscope equipped for fluorescence with a Nikon Intenslight C-HGFI and filters for red (ET Cy3), green (FITC EX 465-495) and blue (ET DAPI) fluorescence. Fluorescence micrographs were taken with a Nikon DS-Ri1 camera using the software NIS-Elements D 3.0. Minor adjustments of brightness and contrast were applied to the entire image for publishing, using Adobe Photoshop Elements V9 (Adobe Systems Incorporated).

The quantification of NKp46^+^ cells followed a determined protocol (Fig. S1) using NIS-Elements D3.0 Annotations and Measurements. Six areas including a small bronchi or bronchioles and the surrounding loose connective tissue and omitting the bronchial or bronchiolar lumina from each of the four lung lobes were defined manually and automatically measured by the software. The NKp46^+^ cells were then defined as showing a clear fluorescence and having a form indicating a cell, and cells that were within the demarcated area or on the line of demarcation were counted. The numbers of NKp46^+^ cells per 0.1 mm^2^ were calculated. Areas both with and without virus were included in the examination. In total, 95 areas from control animals were analysed and compared to similar areas from infected animals. From infected animals, a total of 60 areas without virus and 36 areas with virus from day 1 pi, 81 areas without virus and 13 areas with virus from day 2 pi and 28 areas without virus and 68 areas with virus from day 3 pi were investigated.

From each area three separate micrographs (magnification 200x) were taken with the respective filters to identify influenza A virus NP (red), NKp46 (green) and cytokeratin (blue). The settings of the Nikon intenslight lamp and NIS-Elements D3.0 software were kept constant for the micrographs of NKp46, but minor modifications were made to optimize the micrographs of influenza A virus NP, due to strong variation of quantity of antigen, and of the cytokeratin micrographs due to varying staining intensity. The three separate micrographs were merged to be able to identify influenza A virus NP in cytokeratin^+^ epithelial cells and the NKp46^+^ cells in the same micrograph.

### Binding of NKp46 immunoglobulin fusion protein to influenza virus infected cells

MDCK cells were cultivated in MEM (PAA) supplemented with 10% (v/v) heat-inactivated FBS, 2 mM stable glutamine, 100 IU/ml penicillin and 0.1 mg/ml streptomycin at 37°C. For infection, confluent cells in T25 tissue flasks were inoculated with 180 PFU/ml of the 2009 pandemic H1N1 influenza virus (isolate A/California/07/2009) in medium without serum for 1.5 hours at 34°C. Afterwards, inoculum was removed and cells were cultivated in medium without serum supplemented with 1 µg/ml trypsin for two days at 34°C. Non-infected MDCK cells were treated in an analogous manner.

For the NKp46-binding assay, cells were detached by trypsinization and resuspended in PBS containing 2% FBS. Cells were left on ice overnight for recovery from trypsin-treatment and incubated with different concentrations (40 µg, 20 µg, 10 µg and 5 µg) of a recombinant fusion protein containing the extracellular part of porcine NKp46 and the hinge and Fc part of murine IgG2b [22] for two hours on ice. In addition, cells were stained with anti-H1 mAbs (IgG1, clone C102, AbD Serotec). Anti-H1 mAbs were added for the last 30 min of the NKp46-Ig incubation step to overcome potential competitive effects in the binding to HA antigen. Goat anti-mouse IgG1-PE (Southern Biotech) and Goat anti-mouse IgG2b-AlexaFluor 647 (Invitrogen) antibodies were used as secondary reagents. Mouse IgG1 irrelevant mAbs (clone NCG01, Dianova) were used to control the H1-specific mAb. Cells were analysed by flow cytometry on a FACSCanto II (BD Biosciences). Data for at least 3×10^4^ cells were recorded and data was analysed with FACS-Diva (Version 6.1.3, BD Biosciences) and FlowJo software (Version 7.6.3., Tree Star).

### Analysis of IFN-γ and TNF mRNA by rRT-PCR in lung tissue

Lung mononuclear cells from all infected animals from the second study were analysed for gene expression of IFN-γ and TNF and compared to that of four control pigs. The control pigs were aged matched with the pigs from the study and purchased from a high health status herd. All pigs were negative for influenza A virus determined by rRT-PCR from nasal swabs [40]. Control pigs were euthanized and mononuclear cells were isolated from the lungs and frozen by the same method as described above.

Total RNA was isolated from frozen mononuclear cells from the lungs using RNeasy Mini Kit (Qiagen) according to the manufacturer's protocol. Amplification and detection of IFN-γ (forward primer: 5′ TTCAGCTTTGCGTGACTTTG-3′. reverse primer: 5′-AAGAAAAGAGGTCCACCATTAGG-3, probe: 5′-TexasRed-GCTCTTACTGCCAGGCGCCCBHQ2-3′), TNF (forward primer: 5′-CCCCTGTCCATCCCTTTATT-3′, reverse primer: 5′-ACACATCCCTGAATCCCTGA-3′, probe: 5′-6FAM-ATGAGGGGCTGGGGACTGGG-BHQ1-3′) and the household gene GADPH (forward primer: 5′-GTTCCACGGCACAGTCAAG-3′, reverse primer 5′- CATGGTCGTGAAGACACCCAG-3′, 5′-probe 5′-6FAM-CGGAGAACGGGAAGCTTGTCA-3′) mRNA was performed on a Stratagene Mx3500P using the Qiagen One-Step RT-PCR kit (Qiagen). The RT step condition was 30 min at 50°C followed by 15 min at 95°C. A three-step PCR cycling protocol was used as follows: 45 cycles of 94°C for 10s, 55°C for 20s and 72°C for 10s. Expression of each target gene was compared to that of the household gene and the Ct values of each infected animal were compared to the mean Ct value of the controls as previously described [41].

### Statistical analysis

Statistical analyses were performed using JMP V9 (SAS Institute Inc) and Graph Pad Prism V6 (GraphPad Software). Differences among groups were assessed by the Mann-Whitney test. When three groups or more were present, each pair of groups was compared individually. In some figures, statistical difference between groups is displayed by giving these groups different letters, while groups with the same letter does not differ significantly. Box plots show median values, the 25th and 75th percentiles and the lowest and highest values. Statistical analysis was performed on results from groups with four or more observations only.

## Results

### Influenza A(H1N1)pdm09 virus infection causes bronchointerstitial pneumonia in pigs

To identify the most relevant time period to study NK cells, pigs were experimentally infected with influenza A(H1N1)pdm09 and pairs of infected and control animals were euthanized and sampled 1−8 days pi. All infected animals showed mild clinical signs of influenza [25] on day 1−3 pi and infection was confirmed by detection of viral RNA by rRT-PCR in lung tissue samples (data not shown). At post-mortem examination, macroscopic (Fig. 1A) and histological changes (Fig. 1B) typically associated with influenza [25] were found restricted to the respiratory tract and associated lymphoid tissue. There was a peak in severity and extension of macroscopic pulmonary changes on day 3 pi (Fig. S2). Histological changes included moderate bronchointerstitial pneumonia and necrotizing bronchiolitis (Fig. 1B), and less frequently, lymphocytic rhinitis and tracheitis. The extension of the histological changes increased from day 1 to 3 pi. From day 4 pi, the number of affected bronchi and bronchioles gradually decreased and the epithelia showed sign of regeneration. Influenza A virus NP was detected by immunohistochemistry (Fig. 1C) in the epithelial cells of both the turbinates and trachea and in the bronchi and bronchioles. Positive pneumocytes could be found in areas close to infected bronchioles. Viral NP was also detected in macrophages in respiratory lymph nodes (data not shown). The highest number of infected cells was observed in the lungs at day 1 pi with a progressive reduction throughout the infection.

Blood was collected daily from all remaining pigs, and the NK cells defined as CD3^−^CD8α^+^NKp46^+^ and CD3^−^CD8α^ +^NKp46^−^ cells in PBMC were analysed by flow cytometry. The percentages of NK cells varied between animals, and there was also some day to day variation in the same animal, especially in the control group. During the first three days pi, there was a decrease in NKp46^+^ cells in infected animals (median  =  4.2%, range  =  1.3−12% of lymphocytes) compared to the controls (median  =  6.1%, range  =  2.3−14% of lymphocytes), but the decrease from one day to the other was not significant. NK cell levels in liver, spleen and all lymph nodes investigated were similar in control and infected animals (data not shown).

### A reduction in NKp46^+^ NK cells in the blood of influenza infected pigs was confirmed in a second experiment

Since the histological findings showed that the lung infection was regressing from day 4 pi, the first three days pi was considered as the most relevant time period to study the early involvement of NK cells. A second experiment was performed, including more animals on each sampling day. Lung lesion score [37] was compared between the two experiments (Fig. S2). The median score of the infected animals in the second experiment was higher compared to the animals in the first study, but the differences were not significant due to low numbers of animals. The histological changes, viral distribution as determined by immunohistochemistry and the presence of virus in lung tissue samples as measured by rRT-PCR were similar between the two experiments (data not shown).

**Figure 2:**
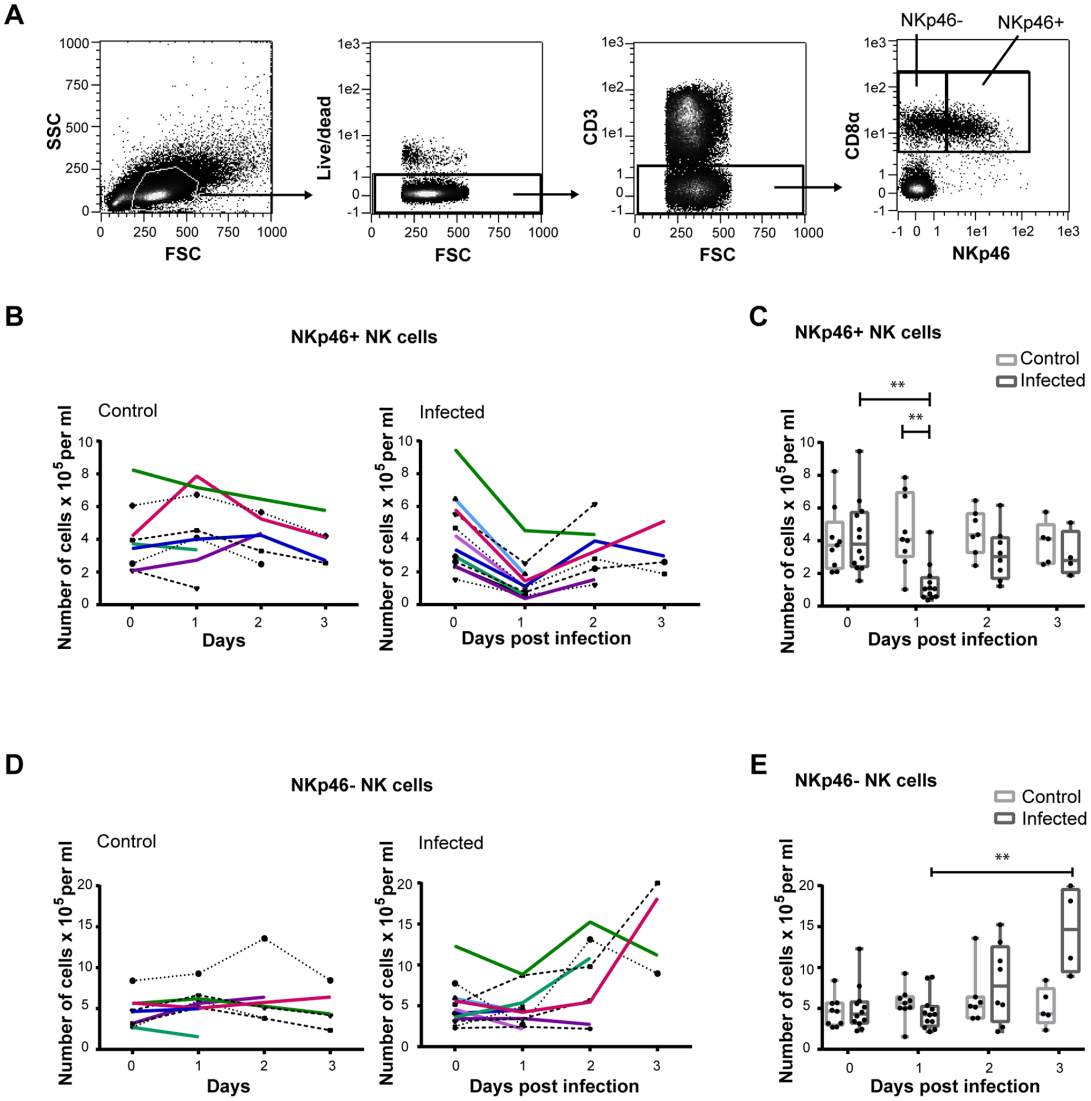
NK cell numbers in the blood of influenza infected pigs. Blood was taken from influenza A virus infected (*n* = 12) and control pigs (*n* = 9) on day 0−3 pi in a second experiment. (**A**) Isolated PBMCs were analysed by flow cytometry and live CD3^−^ lymphocytes were gated as NKp46^−^ or NKp46^+^ NK cells according to CD8α and NKp46 expression. Plots are taken from a representative control animal. Absolute numbers of (**B**) NKp46^+^ NK cells in PBMC were analysed by flow cytometry in control (left) and infected animals (right). (**C**) Results for the NKp46^+^ cells in the two groups were compared on each sampling day. (**D**) NKp46^−^ NK cells in PBMC in control (left) and infected animals (right). (**E**) NKp46^−^ NK cells were compared for the two groups. Each line in (B) and (D) represents one animal. **p≤0.01.

The total numbers of lymphocytes in blood were reduced in the infected animals on the first day after infection (Fig. S3) as assessed by the number of CD45^high^ cells in a defined volume of whole blood. A similar reduction has also been described in cases of human influenza [6], [7]. Live CD3^−^CD8α^+^ lymphocytes were gated as NKp46^−^ or NKp46^+^ NK cells for further analysis (Fig. 2A), and the proportions of NKp46^−^ and NKp46^+^ cells in the blood of individual animals were similar to earlier reports [22]. There was a drop in NKp46^+^ NK cells on day 1 in all infected animals with a subsequent increase on day 2 pi, both in absolute numbers (Fig. 2B and C) and in percentage of lymphocytes (median  =  3.7%, range  =  1.7−12% on day 0; median  =  1.4%, range  =  0.5−6.8% on day 1; median  =  3.0%, range  =  1.4−5.9% on day 2). No reduction was seen in NKp46^−^ NK cells in infected pigs, but rather an increase was observed on day 2 and 3 pi (Fig. 2D and E).

To investigate cell activation and proliferation, PBMCs were additionally stained for CD25 and Ki-67 expression, respectively, but no differences in the expression of these markers were found in NK cells (data not shown). No differences in percentages of NK cells among lymphocytes were seen between control and infected animals in liver or lymph nodes (data not shown).

### Influenza A virus infection leads to up regulation of NKp46 on NK cells in the lungs

**Figure 3:**
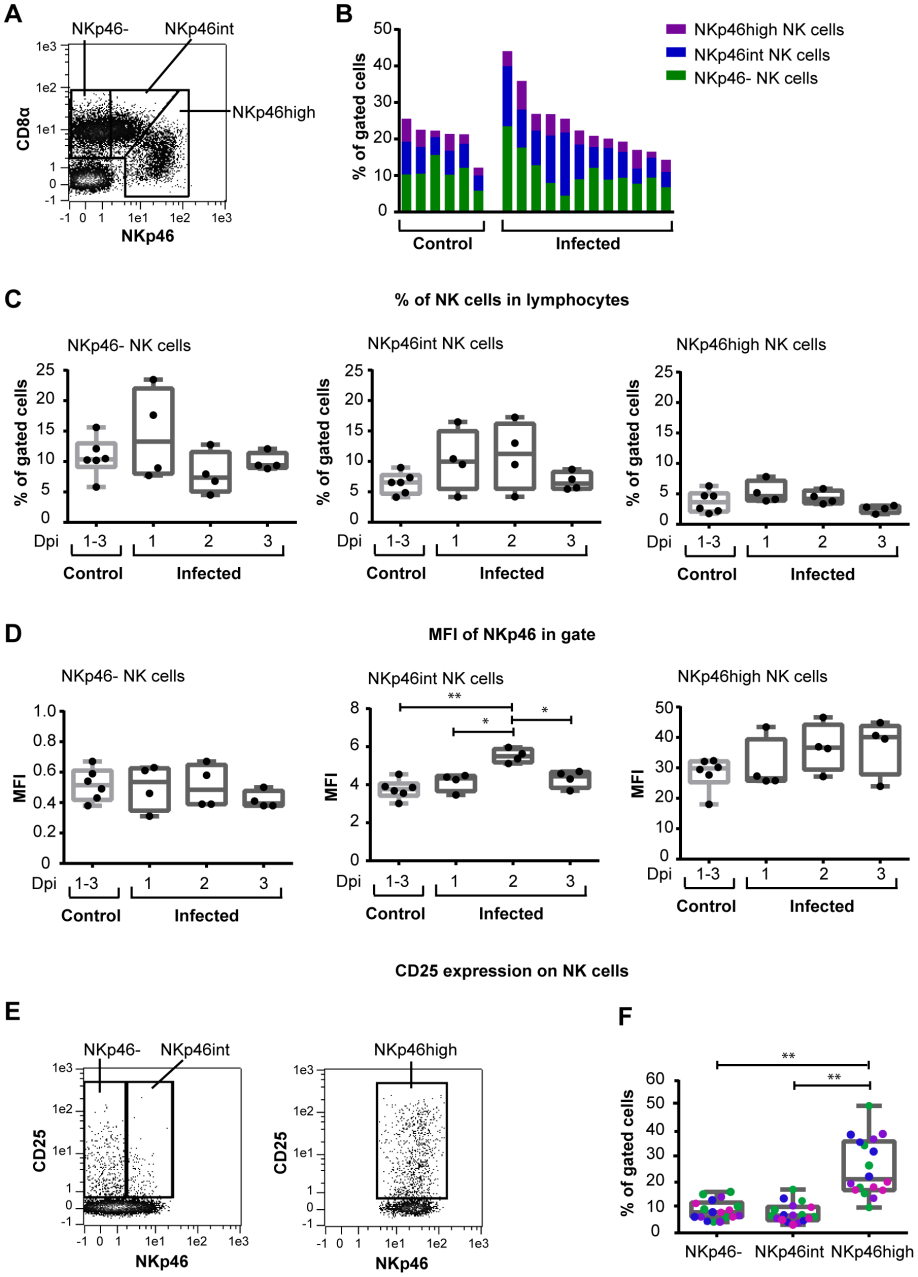
Percentages of NK cells and expression of NKp46 and CD25 in lung tissue. Mononuclear cells were isolated from lung tissue of pigs infected with influenza A virus (*n* = 12) and control animals (*n* = 6) during the first 3 days pi and analysed by flow cytometry. (**A**) Live CD3^−^ lymphocytes were gated as described in Fig 2. NK cells were gated according to CD8α and NKp46 expression and defined as NKp46^−^, NKp46^int^ or NKp46^high^ cells. Plot shown is from a representative control animal. (**B**) Proportions of NKp46^−^ (green), NKp46^int^ (blue) and NKp46^high^ (purple) NK cells in individual animals, shown as percentages of gated cells in lymphocytes. (**C**) Percentages of NKp46^−^ (left), NKp46^int^ (middle) and NKp46^high^ (right) NK cells among lymphocytes. (**D**) Median fluorescence intensity (MFI) in the NKp46^−^ gate (left), the NKp46^+^ gate (middle) and in the NKp46^high^ gate (right) are shown. (**E**) CD25^+^ cells were gated in the NKp46^−^ and NKp46^int^ NK cells (left) and in the NKp46^high^ NK cells (right). Plots shown are from a representative control animal. (**F**) The percentages of CD25^+^ cells in each gate were calculated in control animals (green), infected animals from day 1 (purple), day 2 (blue) and day 3 (pink) pi. *p≤0,05, **p≤0.01.

In the second experiment, mononuclear cells were isolated from lung tissue and analysed by flow cytometry. In the lungs, the CD8α^−/dim^NKp46^high^ population, previously described in swine spleen and liver [22] was found. Thus, the NK cell populations were defined as NKp46^−^, NKp46^int^ and NKp46^high^ NK cells in lung tissue (Fig. 3A). The distribution of the different NK cell populations in individual animals were as shown in Figure 3B.

The percentages of NKp46^−^ NK cells were increased in two of the four infected animals sampled on day 1 pi compared to the control animals, while the percentage of NKp46^high^ NK cells exceeded that of the controls in one infected animal on day 1. The greatest differences were found in the NKp46^int^ NK cells, where three animals on day 1 and three animals on day 2 had higher percentages of NKp46^int^ cells than the control animals (Fig. 3C). However, there were no significant differences between the groups. Nevertheless, there was an increase in the NKp46 expression on NKp46^int^ NK cells, evaluated as median fluorescence intensity (MFI), in the infected animals on day 2 compared to control animals (Fig. 3D). Increased NKp46 expression was also evident on the NKp46^high^ NK cells in several of the infected animals, although the differences between groups were not significant (Fig. 3D).

The mononuclear lung cells were also stained for CD25 (Fig. 3E) and Ki67 (data not shown). No significant increase in the expression of either of these markers was found in infected animals compared to the controls in any of the three NK cell populations. However, a higher percentage of the NKp46^high^ cells were found to express CD25 compared to the NKp46^−^ and NKp46^int^ NK cells in the lungs of both control and infected animals (Fig. 3F).

### Influenza virus infected areas of the lungs were associated with more NKp46^+^ cells

**Figure 4:**
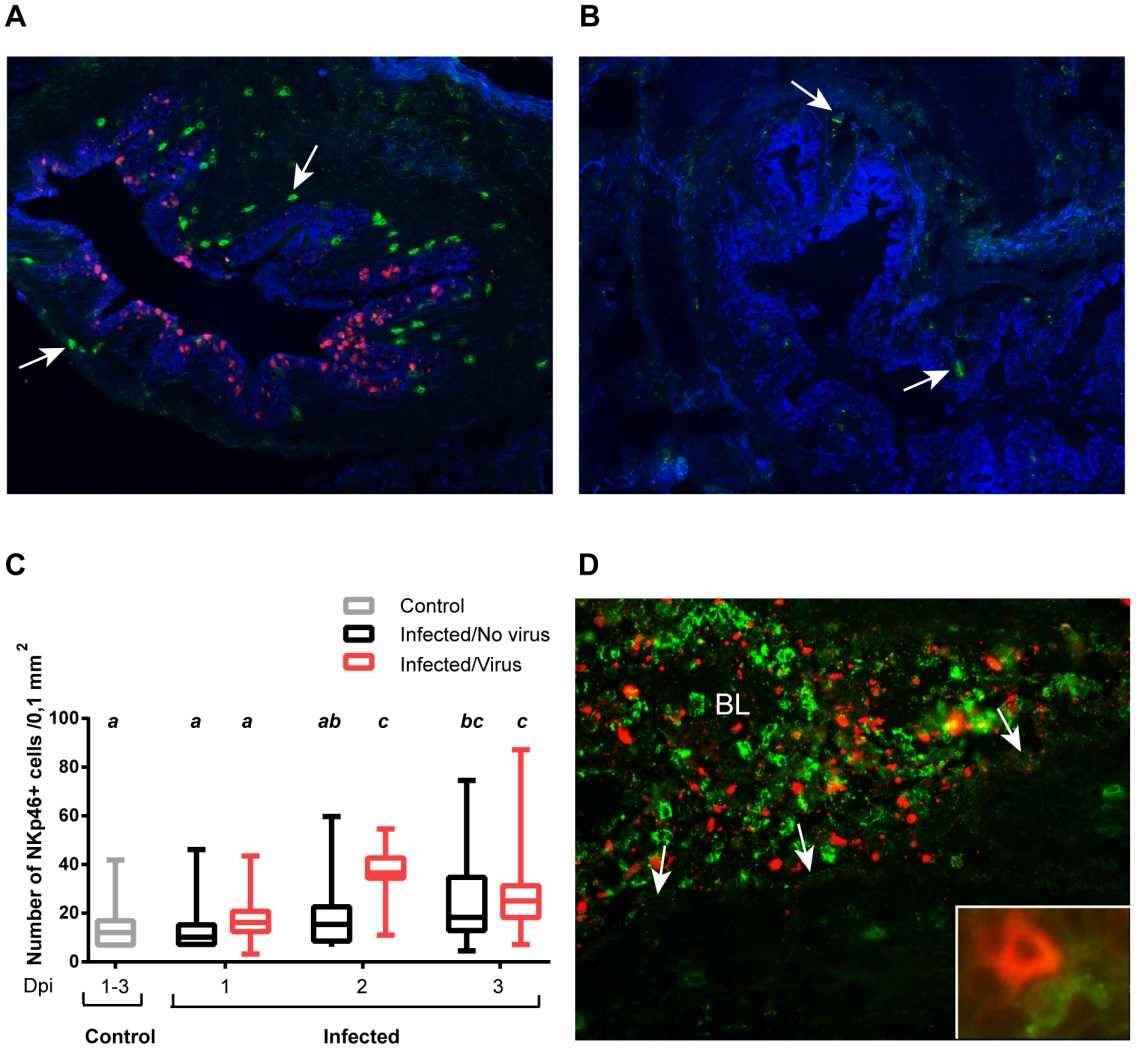
NKp46^+^ cells in the lungs of influenza virus infected pigs. Lung tissue sections from pigs infected with influenza A virus and control pigs were stained with immunofluorescence markers for cytokeratin (blue), NKp46 (green) and influenza A virus NP (red). NKp46^+^ cells were counted in areas were influenza A virus NP was (**A**) detected and (**B**) not detected. Representative pictures taken from the same animal on day 1 pi are shown. Arrows point at NKp46^+^ cells. Immunofluorescence staining, 200x. (**C**) Plot shows number of NKp46^+^ cells per 0,1 mm^2^ in sections (*n* = 24 per animal) from control animals (*n* = 6) and in areas with and without virus in infected animals (*n* = 4 per day) calculated as described in *Material and Methods*. Groups with different letters differ significantly (*p*≤0.05). (**D**) NKp46^+^ cells in the lumen of a bronchus (BL). Arrows point at the epithelial lining. Representative picture of luminal exudate, taken from an infected animal on day 2 pi. Insert shows NKp46^+^ and influenza A virus NP^+^ cell in the lung tissue of an infected animal on day 1 pi. Immunofluorescence staining, 400x.

In order to assess the numbers and distribution of NKp46^+^ cells in lung tissue, sections were stained for influenza A virus NP, NKp46 and cytokeratin 8. The distribution of influenza A virus NP (Fig. 4A) was as shown earlier in Figure 1C. The majority of NKp46^+^ cells were found in the lamina propria of bronchi and bronchioles, in the surrounding connective tissue and in the interalveolar septa, while few NKp46^+^ cells were seen in the epithelial lining (Fig. 4A and B). The distribution of NKp46^+^ cells in the tissue was similar in infected and control animals, and these cells were seen as both scattered cells and in clusters.

As the influenza A virus NP^+^ cells had a multifocal distribution in the lung tissue, areas surrounding small bronchi and bronchioles with and without influenza virus NP^+^ cells were selected to enumerate NKp46^+^cells and compared against similar areas from control animals (Fig. S1). In infected animals, lung areas with influenza virus NP^+^ cells contained more NKp46^+^ cells (Fig. 4A) than areas where influenza virus NP could not be detected (Fig. 4B). There was a peak in the number of NKp46^+^ cells on day 2 pi in areas with virus, while the number of NKp46^+^ cells in areas without virus gradually increased from day 1 to 3 pi (Fig. 4C). Similar results were obtained from all lung lobes sampled. In animals that had cellular exudate in the lumen of bronchi and bronchioles, a high number of NKp46^+^ cells were present in the exudate (Fig. 4D). In several areas of the lung tissue, NKp46^+^ and influenza virus NP^+^ cells were found in immediate proximity to each other, and in some cases these cells had a direct contact (Fig. 4D).

### NKp46^+^ cells in the lungs are not infected with influenza virus and do not undergo apoptosis

**Figure 5:**
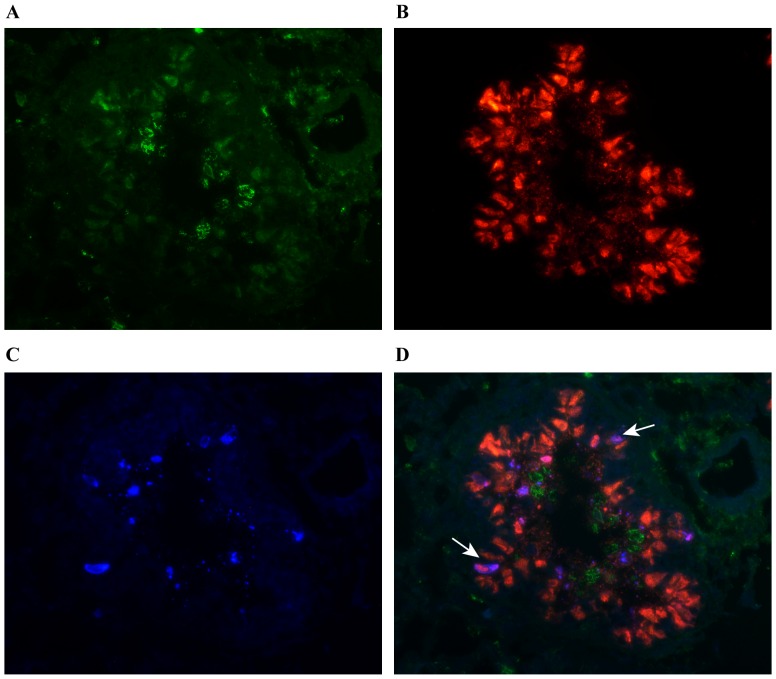
Staining for apoptosis in the lungs. Lung tissue sections from animals infected with influenza A virus (*n* = 12) were stained with immunofluorescence markers against (**A**) NKp46(green), (**B**) influenza A NP(red) and (**C**) the apoptosis marker caspase-3(blue). (**D**) Overlay displaying simultaneously influenza A virus NP^+^ and caspase-3^+^ cells as purple (arrows). Representative of virus infected bronchiole at day 1 pi. Immunofluorescence staining, 400x.

Influenza virus propagation leads to apoptosis of infected epithelial cells through activation of caspase-3 [42], and it has been suggested that induction of apoptosis in NK cells in lung tissue may be an important escape mechanism for influenza A virus [6], [8]. Samples from lung tissue were therefore stained for NKp46 (Fig. 5A) and influenza A virus NP (Fig. 5B) in combination with caspase-3 (Fig. 5C). No cells in the lung tissue were stained double positive for influenza A virus NP and NKp46, indicating that virus did not replicate in the NKp46^+^ cells. Abundant caspase-3^+^ cells were found in the epithelial lining of bronchi and bronchioles and most of these cells were infected with influenza virus (Fig. 5D). However, no double positive NKp46 and caspase-3 stained cells were found.

### Elevated levels of TNF, but not IFN-γ in lung tissue

**Figure 6:**
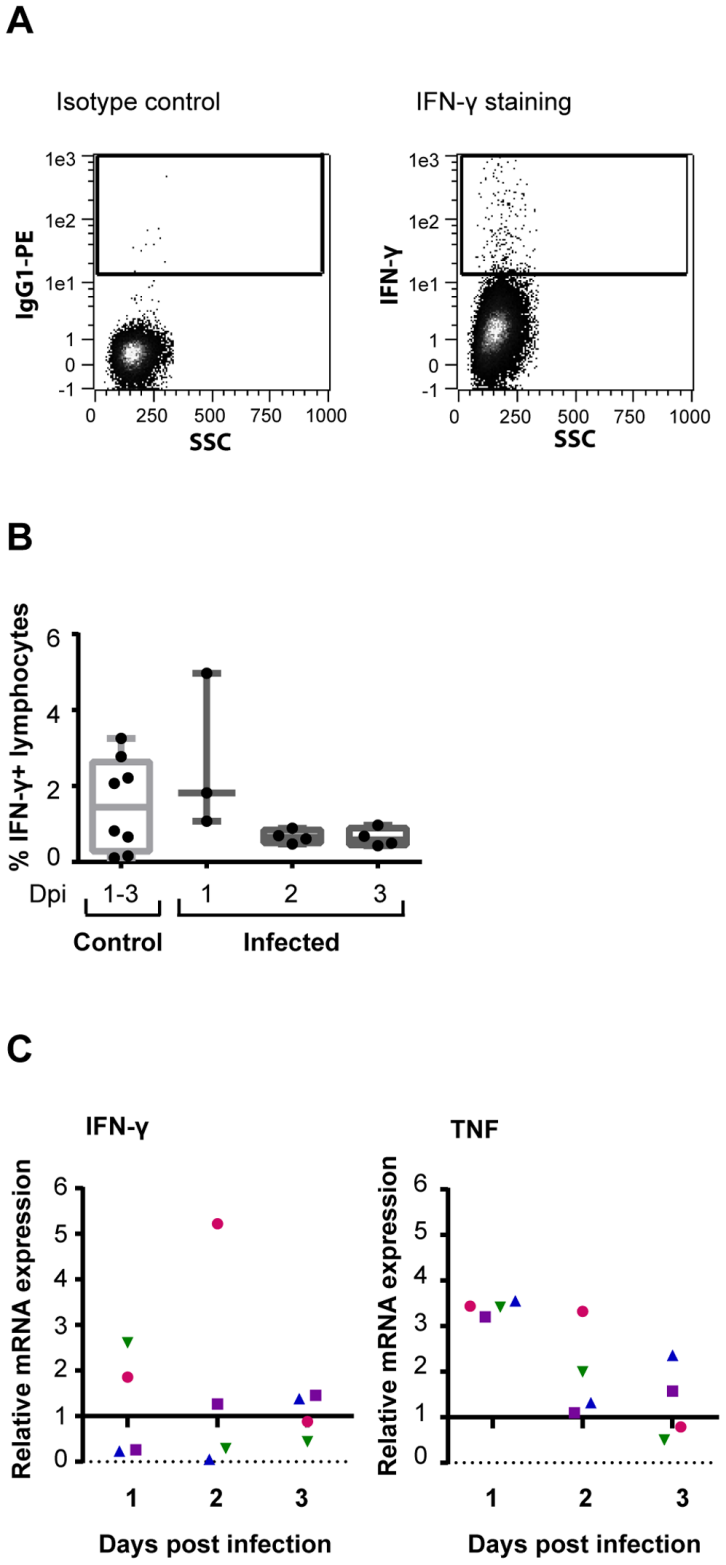
Detection of IFN-γ and TNF in lung tissue. (**A**) Intracellular IFN-γ was analysed in lung mononuclear cells from influenza A virus infected animals and control animals by flow cytometry. IFN-γ ^+^ cells were gated among live lymphocytes. Plots show representative isotype control (left) and IFN-γ staining (right) from the same infected animal on day 3 pi (**B**) Percentages of IFN-γ^ +^ cells obtained by flow cytometry in control animals (*n* = 8) and infected animals (*n* = 4 per day). Data from one animal at day 1 is missing due to too few cells isolated. (**C**) Gene expression for IFN-γ and TNF mRNA in infected animals (*n* = 4 per day) was calculated as relative values to the household gene GADPH and to mRNA levels of the target gene in a control group (*n* = 4). Each symbol represents one infected animal, different symbols represents animals sacrificed the same day. Values above 1 indicate an up regulation, whereas values below 1 indicate a down regulation of the target gene.

To investigate the intracellular protein levels of IFN-γ in lung tissue cells, isolated lung mononuclear cells were stained with an antibody against IFN-γ and analysed by flow cytometry (Fig. 6A). The results showed some day to day variation in both control and infected animals and no clear differences in IFN-γ production between the infected and control group could be detected (Fig. 6B). A greater portion of the IFN-γ^ +^ cells were CD3^+^, but IFN-γ^ +^CD3^−^ cells were also observed. Some of the IFN-γ^ +^CD3^−^ cells expressed NKp46, but this population did not differ between control and infected animals (data not shown). IFN-γ production was also investigated on mRNA level by rRT-PCR detection using specific primers for IFN-γ mRNA in isolated lung mononuclear cells from infected animals. Consistent with flow cytometric analysis, the relative expression of IFN-γ mRNA varied between the individual infected animals and no difference between infected and control groups were found (Fig. 6C). Expression of TNF mRNA was also investigated by rRT-PCR and the results showed an increase in TNF levels in all infected animals relative to healthy pigs on day 1 and 2 pi (Fig. 6C).

### The NKp46 receptor binds to influenza virus infected cells

**Figure 7:**
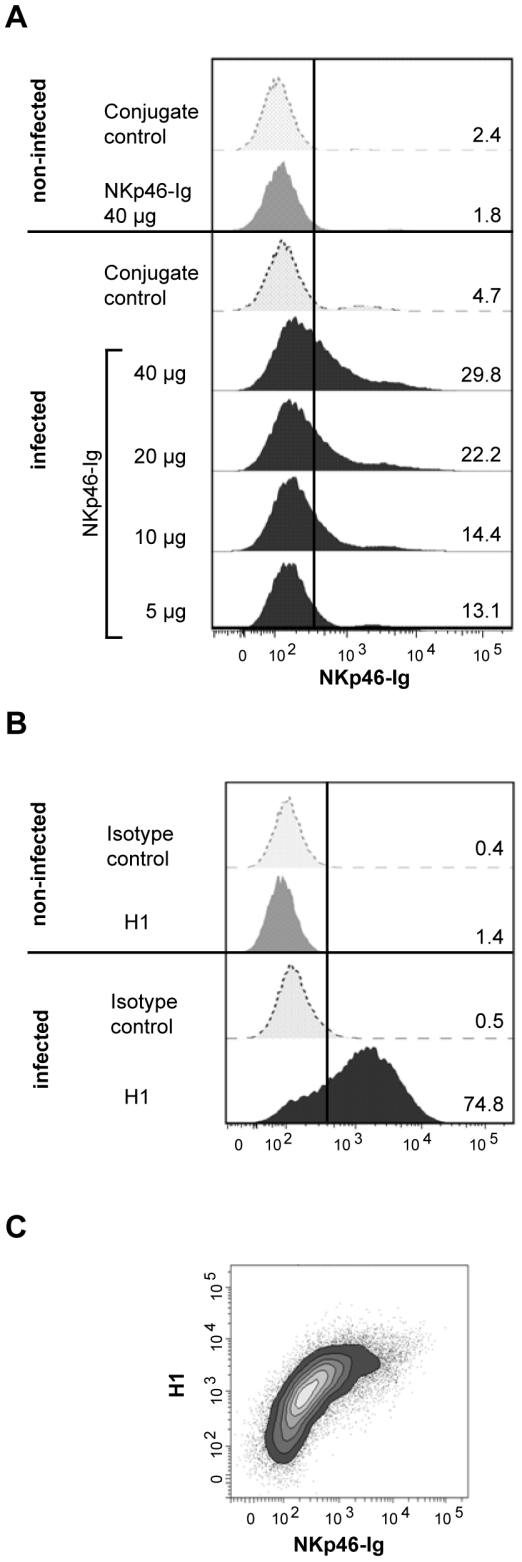
Binding of porcine NKp46 Ig fusion protein to of influenza A virus infected MDCK cells analysed by flow cytometry. Cells were gated according to forward/side scatter characteristics. (**A**) Histograms show binding of different concentrations of NKp46-Ig to infected cells. Percentages of positively stained cells are indicated. (**B**) Infected as well as non-infected MDCK were stained with anti-H1 mAbs. Corresponding isotype-matched irrelevant mAbs and secondary antibodies only served as controls. (**C**) Double staining for H1 and the highest concentration of NKp46-Ig are shown on influenza infected cells. Results are representative of four independent experiments.

The *in vivo* studies indicated a role for NKp46^+^ cells in the lungs of influenza A virus infected pigs. Therefore, an *in vitro* binding assay was carried out to determine whether porcine NKp46 binds to influenza A virus infected cells as has been shown for human NKp46 [3], [4]. Recombinant porcine NKp46-Ig fusion protein showed dose-dependent binding to influenza A virus infected cells (Fig. 7A). No binding of NKp46-Ig on uninfected cells was observed. As expected, a clear H1 expression could be observed on infected cells, whereas no expression was observed on non-infected MDCK cells (Fig. 7B). When using anti-H1 mAbs and NKp46-Ig in combination, a distinct co-staining could be observed on influenza A infected cells (Fig. 7C).

## Discussion

Interactions between NK cells and influenza A virus in the lungs during the early phase of infection are still poorly understood. *In vitro* studies have shown that human NK cells can be activated by binding of viral HA to NKp46, leading to direct killing of infected cells [3], [4], [14], [43]. Conversely, blocking of this receptor results in reduced NK cell activation [14], [44]. The present study provides evidence that porcine NKp46 also binds to influenza A virus infected cells, suggesting that this receptor has a defined role in influenza virus infections in several species.

A reduction in NKp46^+^ NK cells was seen in the blood of infected pigs shortly after infection. As demonstrated by complete blood counts, there was a general decrease in lymphocytes. Hence, the reduction of NKp46^+^ NK cells was not unique, but did differ from NKp46^−^ NK cells, which increased in numbers. A similar reduction of CD3^−^CD56^+^ NK cells has been observed in humans with influenza, leading to the hypothesis that NK cells are recruited to the lungs to participate in the defence against the influenza virus [6], [7]. In this study, immunofluorescence staining of lung tissue sections from pigs infected with influenza virus demonstrated a clear difference in the numbers of NKp46^+^ cells in areas infected with virus, compared to uninfected areas from the same animal. There was also an overall increase in the number of NKp46^+^ cells in lung sections from the infected animals compared to control animals. It was not possible to separate the NKp46^int^ and the NKp46^high^ NK cell population in the stained tissue sections since no marker has been found to be exclusively expressed by either populations [23] and it is likely that both the NKp46^int^ and the NKp46^high^ NK cells are identified as NKp46^+^ cells. NKp46^+^ cells were found to co-localize with influenza virus NP^+^ cells and in some cases the cells seemed to have contact. This could potentially represent NK cells bound to influenza infected cells as demonstrated by NKp46 binding to infected cells *in vitro*, but other receptors may also account for this contact and need to be further elucidated. These results support the hypothesis that NK cells are recruited to the influenza virus infected parts of the lungs.

Flow cytometric analysis showed only a minor increase in the percentage of NK cells in lung tissue. As other cell types are also recruited to the lungs following influenza virus infection, cell numbers measured as percentages of the mononuclear cell population is likely to underestimate the absolute number of NK cells in lung tissue. Also, since the virus has a multifocal distribution in the lungs, the amount of NK cells will be influenced by the number of microscopic lesions in the sample. This could explain some of the individual differences seen between infected animals in the flow cytometric analysis. The earlier reports of NK cell numbers in lung tissue following virus infection as measured by flow cytometry in swine are diverse. One study found an increase in NK cells in bronchoalveolar lavage fluid from swine starting at 2 days pi [33], while another study found a decrease in NK cells in lung tissue on day 3 pi and in bronchoalveolar lavage fluid on day 6 pi [29].

As *in vitro* studies have shown that influenza virus is able to infect human NK cells, leading to increased detection of apoptotic markers [8] it has been suggested that the reduced number of NK cells in the blood of influenza virus infected humans is caused by apoptosis of infected NK cells in lung tissue causing a drainage of NK cells to the lung [5], [6]. In the terminal stages of fatal influenza, humans have diminished numbers of NK cells in lung tissue [5], [9]. However, since the lung tissue of human patients was sampled in the terminal stage of the infection, it is impossible to determine whether the NK cells had been present in the lung at earlier time points. In the present study, neither cells that were double positive for NKp46 and the apoptosis marker caspase-3, nor NKp46 and influenza A virus NP, were identified. This indicates that infection and apoptosis of NKp46^+^ cells is not an important escape mechanism for the influenza virus in the early phase of the infection in swine. NKp46^+^ cells were observed in the lumen of bronchi and bronchioles of influenza virus infected animals, and the number of NK cells entering the exudate could influence NK cell numbers in the lung tissue and blood.

A population of NKp46^high^ NK cells was found in the lungs of both control and infected animals. This population was also found in liver and spleen in the current study (data not shown) and has been described earlier [22]. Increased expression of NKp46 has been linked to activation of NK cells in both swine and humans [23], [45] and in line with this, a higher percentage of the lung NKp46^high^ cells in this study expressed the activation marker CD25 compared to NKp46^−^ and NKp46^int^ NK cells. A higher expression of CD27 has also been found on this population in spleen [23]. We hypothesise that the NKp46^high^ population in the lungs represents a more mature or activated NK cell population, in line with what has been shown for NKp46^high^ NK cells in spleen [23]. Since NK cells are primarily produced in the bone marrow and released to the blood in steady state [46] and during influenza infections [12], we speculate that the NKp46^high^ NK cell population may have originated from the bone marrow and reached the lungs through the blood, before maturing or being activated in the lungs.

An increased NKp46 expression was also seen in the population of NKp46^int^ cells following influenza A virus infection. It has been shown earlier that porcine NK cells may up-regulate NKp46 in response to *in vitro* cytokine stimulation [22]. This may well be the case *in vivo*, and is also most likely caused by activation of the cells [23], [45]. There was a tendency of increased NKp46 expression in the NKp46^high^ NK cells as well, although not significant. This could imply that mainly the cells expressing the NKp46 receptor respond to the influenza A virus infection. Furthermore, these results suggest that the NKp46^int^ cells are the main responders to the infections and therefore might be responsible for the greatest part of the increase in NKp46^+^ cells seen in the immunofluorescence staining of lung sections.

A decrease in the numbers of NKp46^+^ NK cells in the blood of infected pigs was observed in both experiments, but a significant reduction was only seen at day 1 pi in the second study. In the first study, the pigs were inoculated with a 1∶1 mixture of two different virus variants, namely A (H1N1) pdm09 222D (aspartic acid) and 222G (glycine). These viruses differ only in the amino acid positioned at 222 (H1 numbering) in the viral surface glycoprotein HA. A more severe infection has been associated with the 222G variant, as compared to when only the wild-type 222D variant is detected [47]. In the second experiment, only the 222G virus variant was used. This resulted in a wider distribution of macroscopic changes. The more pronounced NK cell response seen in the second study indicates that the type of virus and severity of the disease may influence the NK cell response in pigs. In humans, a more marked reduction of NK cell numbers in blood has been observed in severe cases of influenza when compared to mild cases [6]. Different influenza viruses have also been shown to activate NK cells to varying extents *in vitro* [43], [44].

Elevated levels of IFN-γ have been detected in nasopharyngeal secretions of human patients with influenza [9], [19]. Also in swine, elevated levels of IFN-γ in the respiratory tract of influenza infected animals have been reported, but the detection varied over time, indicating the need for more frequent sampling [17], [29]. In the present study, no changes in IFN-γ protein levels or mRNA expression in the lungs could be detected. On the other hand, an increased expression of TNF mRNA was found in lung mononuclear cells from all infected animals on day 1 and 2 pi. TNF is an important antiviral cytokine which has been shown to inhibit influenza viral replication in porcine lung epithelial cells *in vitro* and have greater effect against influenza virus replication than IFN-α and IFN-γ [48]. The levels of TNF in the respiratory tract correlates with pulmonary lesion scores and clinical disease in pigs [17], [18] and with body temperature in humans [19], indicating an important link with the induction of clinical signs [17], [18]. Unfortunately, TNF was only investigated on mRNA level on the total mononuclear cell population and the relative contribution of NK cells cannot be concluded. Future studies should define the relative contribution of NK cells in cytokine production and further elucidate the role of different cytokines during influenza virus infections. It is also possible that the main function of NK cells in influenza A virus infections is cytotoxic killing of infected cells, rather than cytokine production. NK cells have been shown to kill influenza virus infected cells *in vitro* [3], [4], [43]. Furthermore, studies in mice point to an important role for NK cell-mediated lysis in clearing influenza A virus from the lungs *in vivo* [4], [10], but this needs further investigation.

In summary, a decline in NKp46^+^ NK cells was demonstrated in the blood of infected pigs; similar to what is seen for CD3^−^CD56^+^ NK cells in humans, strengthening the idea of the pig as a model for influenza virus infections in humans. The changes in blood were followed by an increase in NKp46^+^ NK cells in influenza virus infected areas of the lungs, providing further insight into the dynamics of NK cells during influenza. The specific increase in NKp46 expression on NK cells expressing the NKp46 receptor, cells in the lung tissue of infected pigs indicates a higher activation status of these cells and suggests a functional role for these cells in the lungs of influenza infected swine. Further studies should address the functional aspect of these cells.

## Supporting Information

Figure S1 Counting of NKp46^+^ cells. Numbers of NKp46^+^ cells per area were counted in lung tissue sections from control animals and influenza A virus infected animals as described in *Material and Methods*. Representative picture of area with virus from infected animal. Immunofluorescence staining, 200x.
(TIF)

Figure S2 Comparison of gross lung lesion score. Lung lesions were compared between the two studies. In the first study, pigs were infected with a mixture of influenza A (H1N1) pdm09 225D and 225G. In the second study, only the 225G variant was used. Macroscopic pathology was evaluated as gross lung lesion score on days 1–5 and 8 pi in the first experiment (*n* = 2 per day) and on days 1–3 in the second experiment (*n* = 4 per day).
(TIF)

Figure S3 Influenza A virus infection causes lymphophenia in pigs**.** Complete blood counts in influenza A virus infected pigs were determined by CD45 staining and flow cytometric analysis of PBMC. (**A**) Lymphocytes were gated as CD45^high^ cells with a low side scatter (SSC) and granulocytes as CD45^dim^ cells with a high SSC according to CD45 expression and SSC. (**B**) Lymphocyte and granulocyte numbers in infected (*n* = 12) and control animals (*n* = 9) were obtained each day until they were sacrificed. *p≤0.05, **p≤0.01.
(TIF)
