# Supplementary figures and images for: Early Responses of Natural Killer Cells in Pigs Experimentally Infected with 2009 Pandemic H1N1 Influenza A Virus
Source: PLoS One. 2014 Jun 23;9(6):e100619. doi: 10.1371/journal.pone.0100619 (PMC4067341; doi:10.1371/journal.pone.0100619)

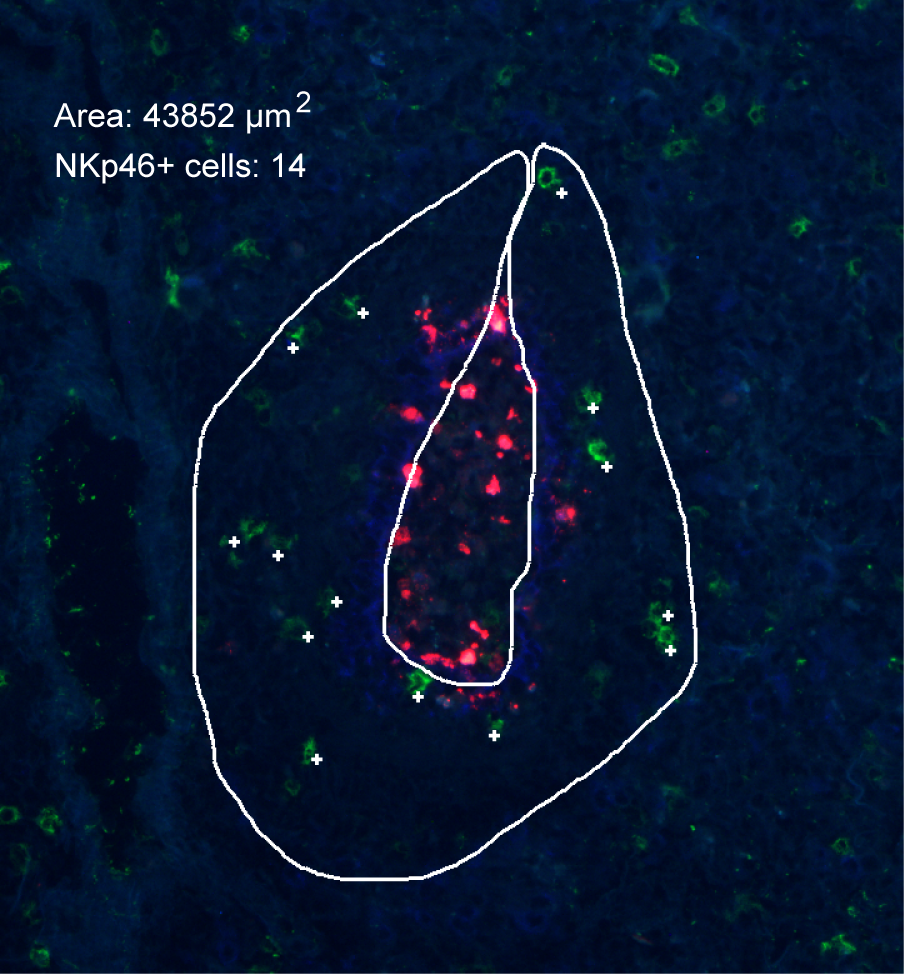

Supplement: Figure S1 — Counting of NKp46+ cells. Numbers of NKp46+ cells per area were counted in lung tissue sections from control animals and influenza A virus infected animals as described in Material and Methods. Representative picture of area with virus from infected animal. Immunofluorescence staining, 200x. (TIF) [file pone.0100619.s001.tif]

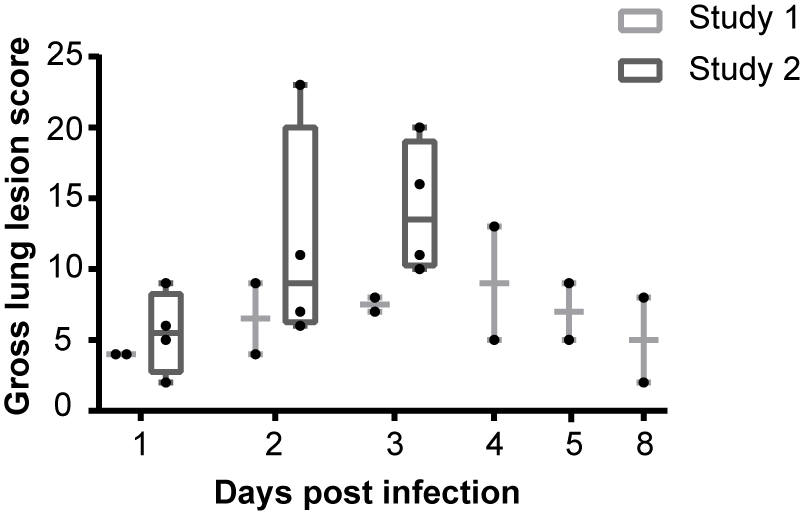

Supplement: Figure S2 — Comparison of gross lung lesion score. Lung lesions were compared between the two studies. In the first study, pigs were infected with a mixture of influenza A (H1N1) pdm09 225D and 225G. In the second study, only the 225G variant was used. Macroscopic pathology was evaluated as gross lung lesion score on days 1–5 and 8 pi in the first experiment (n = 2 per day) and on days 1–3 in the second experiment (n = 4 per day). (TIF) [file pone.0100619.s002.tif]

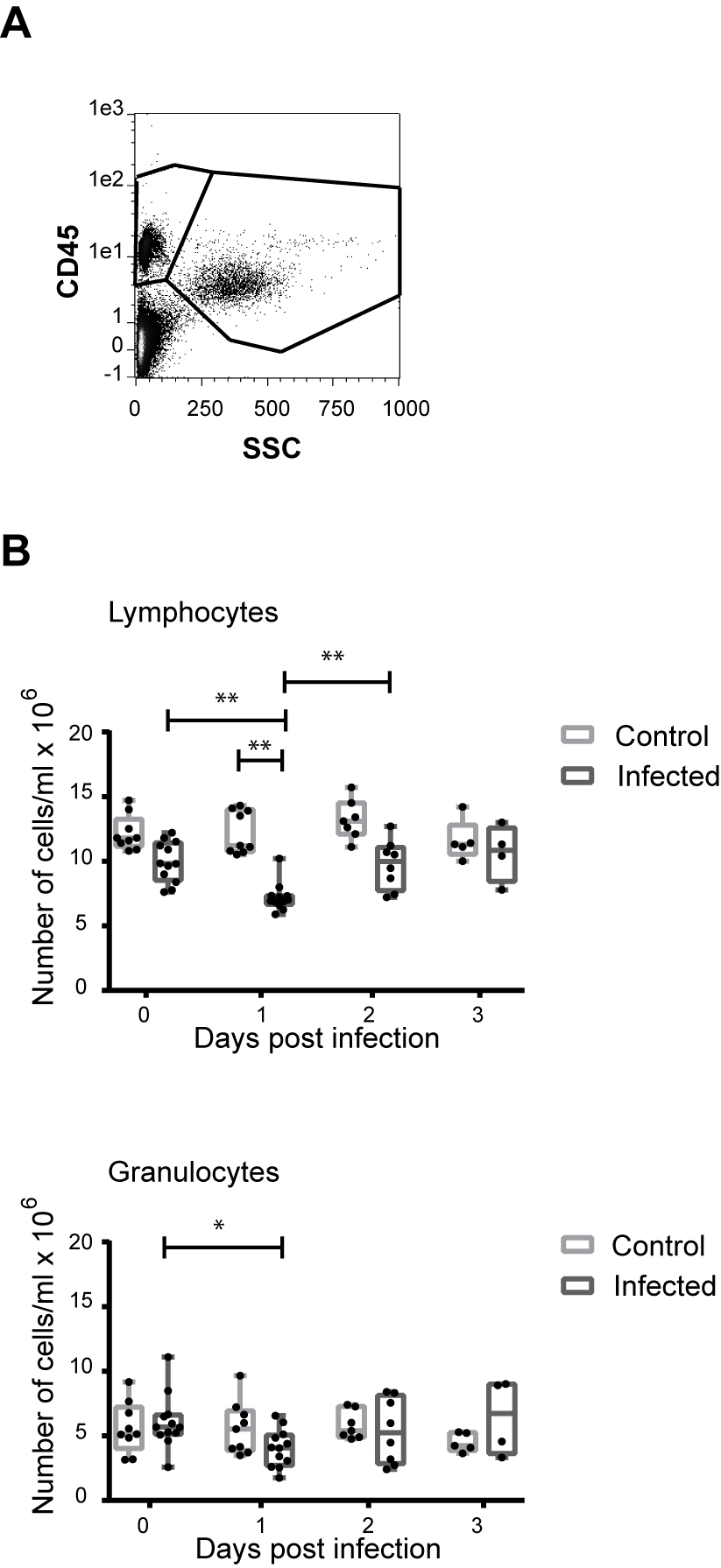

Supplement: Figure S3 — Influenza A virus infection causes lymphophenia in pigs. Complete blood counts in influenza A virus infected pigs were determined by CD45 staining and flow cytometric analysis of PBMC. (A) Lymphocytes were gated as CD45high cells with a low side scatter (SSC) and granulocytes as CD45dim cells with a high SSC according to CD45 expression and SSC. (B) Lymphocyte and granulocyte numbers in infected (n = 12) and control animals (n = 9) were obtained each day until they were sacrificed. *p≤0.05, **p≤0.01. (TIF) [file pone.0100619.s003.tif]
